# Climate sensitivity effects on tropical bat ecology

**DOI:** 10.1016/j.gecco.2026.e04471

**Published:** 2026-10-13

**Authors:** Prateep Duengkae, Luis E. Escobar, Warong Suksavate, Aingorn Chaiyes, Kornsorn Srikulnath, Supaporn Wacharapluesadee, Somchai Panphuangkaew, Kevin J. Olival, Emma V. Willcox, Praewa Mahaphol, Paanwaris Paansri

**Affiliations:** a Special Research Unit for Wildlife Genomics (SRUWG), Department of Forest Biology, Faculty of Forestry, Kasetsart University, Bangkok, 10900, Thailand; b Animal Genomics and Bioresource Research Unit (AGB Research Unit), Faculty of Science, Kasetsart University, Bangkok, 10900, Thailand; c Department of Fish and Wildlife Conservation, Virginia Tech, Blacksburg, VA, 24061, United States; d The International Undergraduate Program in Bioscience and Technology, Faculty of Science, Kasetsart University, Bangkok, 10900, Thailand; e Thai Red Cross Emerging Infectious Diseases Clinical Center, King Chulalongkorn Memorial Hospital, Bangkok, 10330, Thailand; f Department of National Parks, Wildlife and Plant Conservation, Bangkok, 10900, Thailand; g College of Tropical Agriculture and Human Resilience, University of Hawaiʻi at Mānoa, Honolulu, HI, 96822, United States; h School of Natural Resources, University of Tennessee, Knoxville, TN, 37996, United States

**Keywords:** Climatic lag, Climate sensitivity, Emergence time, Guano yield, *Mops plicatus*, Tropical bats

## Abstract

Tropical ecosystems are highly sensitive to climatic variation, yet biological responses to climate fluctuations often emerge after substantial temporal lags. Insectivorous bats are useful sentinels of climatic dynamics due to their high metabolic demands and reliance on weather-sensitive insect prey. We quantified the extent to which weather variation correlated with the short-term behavioral and long-term foraging responses of a large wrinkle-lipped free-tailed bat (*Mops plicatus*) colony in Thailand. As response variables, we used a nine-year dataset (2013–2021) of daily bat evening emergence times and a 22-year dataset (1998–2019) of monthly guano yields. As predictor variables, we used ERA5-Land reanalysis estimates of temperature and precipitation extracted from concentric ring buffers (0–10, 10–20, and 20–30 km) surrounding the bat colony. Sliding-window analysis showed that bat evening emergence time tracked sub-seasonal weather variation at lags of a few days to about three months. Preceding temperature and precipitation were associated with shifts in bat evening emergence timing. Monthly guano yield declined with elevated temperatures at lags of up to three months. Guano yield showed a sustained long-term decline of 56.62% over two decades (seasonal Mann-Kendall Z = −11.585, p < 0.001). Behavioral response alone appeared insufficient to offset the decline in guano yield, which suggests that alternative factors influence guano production, including declines in prey availability, bat abundance, or foraging time. Our results show that increasing temperatures correlated with seasonally contrasting shifts in bat evening emergence time (later in April-May, earlier in September-October) and reduced guano production up to three months after warming periods occurred.

## 1. Introduction

Tropical ecosystems are highly sensitive to shifting climatic patterns (Eguiguren-Velepucha et al., 2016), yet biological responses to weather anomalies are rarely immediate (Chen et al., 2023). Instead, responses of biological systems to weather anomalies are expressed asynchronously with temporal lags that obscure our understanding of global change biology (Wu et al., 2015). Here, “weather” is defined as short-term (daily-to-monthly) variations in temperature and precipitation, while “climate” refers to longer-term trends (Haest et al., 2021; Power et al., 2023). Insectivorous bats are suitable bioindicators of complex eco-climatic dynamics due to their high metabolic demands, reliance on weather-sensitive insect prey, and specific thermal requirements for roosting, foraging, and reproduction (Jones et al., 2009).

In Southeast Asia, bat species such as the wrinkle-lipped free-tailed bat (*Mops plicatus*; Molossidae, formerly *Chaerephon plicatus*; Ruadreo et al., 2025) play a keystone role in both natural and agricultural ecosystems due to their large abundance. As high-altitude aerial foragers, massive colonies of wrinkle-lipped free-tailed bats provide vital biological pest control services. Wrinkle-lipped free-tailed bats depredate migratory swarms of agricultural pests, like planthoppers, before they damage local rice crops (Leelapaibul et al., 2005; Srilopan et al., 2025; Wanger et al., 2014). In Thailand, the wrinkle-lipped free-tailed bat occupies approximately 18 known limestone cave colonies (Ruadreo et al., 2025), and their population has historically been estimated at ~8 million individuals (Boonkerd and Wanghongsa, 2002). Nevertheless, recent wrinkle-lipped free-tailed bat population estimations remain limited, but some argue that their populations show signs of decline (Ruadreo et al., 2025).

Population-genetic evidence indicates that wrinkle-lipped free-tailed bat colonies in Thailand form a single, panmictic population maintained by high gene flow (Ruadreo et al., 2025). Wrinkle-lipped free-tailed bat reproduction is markedly seasonal and bimodal, with two parturition periods each year (primarily in April and October), so that pregnancy and lactation occur twice annually in phase with the monsoon (Furey et al., 2018; Utthammachai et al., 2008). Because insect population dynamics are tightly coupled to weather, such as seasonal rainfall (Novais et al., 2019; Nurul-Ain et al., 2017), wrinkle-lipped free-tailed bats that depredate these insects could be vulnerable to climatic decoupling, i.e., shifting weather and climate patterns that disrupt the synchrony among rain cycles, prey availability, and bat reproduction.

Previous studies have established linkages between environmental variation and bat behavior, physiology, phenology, and demography (Haest et al., 2021; Mundinger et al., 2021; Power et al., 2023; Stapelfeldt et al., 2023). Organisms generally cope with short-term environmental fluctuations through phenotypic plasticity, encompassing behavioral and physiological responses to environmental change (Piersma and Drent, 2003). Nevertheless, when environmental variation exceeds the adaptive capacity of insectivorous bats (Chiroptera), effects could manifest as long-term demographic changes (Festa et al., 2023; Radchuk et al., 2019), an outcome especially likely for organisms whose resources depend on climatic cycles (Nurul-Ain et al., 2017). For instance, bat evening emergence time, a key foraging behavior in insectivorous bats, may be modulated by weather variation and by the physiological burdens of pregnancy and lactation (Ciechanowski et al., 2007; Duvergé et al., 2000; Reichard et al., 2009). Population-level metrics, including colony size and guano yield, may depend on the seasonal productivity of the insect prey base, which is influenced by historical rainfall (Cumming and Bernard, 1997).

Linking the temporal dynamics of weather variation to the behavior and population dynamics of tropical insectivorous bats is critical for anticipating how ongoing climate change will affect their pest-suppression services, yet this link remains poorly resolved, particularly in the tropics, where large-scale monitoring is scarce (Frick et al., 2020; Kerth and Wolf, 2025). We investigated the temporal lags between weather variation and bat ecological signals of daily evening emergence time as a proxy for foraging behavior and monthly guano yield as a proxy for relative abundance. We used data from a well-documented wrinkle-lipped free-tailed bat colony in Thailand. We hypothesized that bat evening emergence time would track short-term weather variation, reflecting behavioral plasticity, whereas guano yield, an integrated measure of foraging output and colony size, would respond to longer-term weather variation.

## 2. Materials and methods

### 2.1. Study site

The study was conducted at a bat cave located at the Khao Chong Phran Non-hunting Area (KCP NHA) in Ratchaburi Province, western Thailand (13.719° N, 99.771° E) (Fig. 1). The primary bat species at this roost is the wrinkle-lipped free-tailed bat (Hillman, 2006). Khao Chong Phran is a limestone outcrop surrounded by rice fields, sugarcane plantations, and human settlements, with elevations ranging from 70 to 180 m a.s.l. The area contains five caves, with the largest cave of wrinkle-lipped free-tailed bat roosts being approximately 150 m long, 50 m wide at its widest point, and 40 m high. This cave also serves as a roosting site for the wrinkle-lipped free-tailed bat, the cave nectar bat (*Eonycteris spelaea)*, and various tomb bat species (*Taphozous* spp.; Hillman, 1999; Leelapaibul et al., 2005). The climate in the area is tropical monsoon, with a mean annual rainfall of 1486 mm, approximately 90% of which occurs during May-October, while the dry season extends from November to April (Leelapaibul et al., 2005).

### 2.2. Spatial scale

To capture spatial variation of surrounding environmental conditions relevant to bat activity and resource availability, we collected weather data from multiple ring buffers (0–10, 10–20, and 20–30 km; Fig. 1a) surrounding the cave (Lintott et al., 2016; Srilopan et al., 2018). The ring buffers were chosen as the radius of environmental influence to span the area over which the wrinkle-lipped free-tailed bat colony forages. GPS-tracked wrinkle-lipped free-tailed bats in Thailand foraged a median maximum distance of 26 km from their roost, with individual maxima of 13–95 km (Srilopan et al., 2025). The innermost 0–10 km ring buffer therefore represents the local scale at KCP NHA, while the 10–20 and 20–30 km ring buffers capture progressively broader regional conditions.

### 2.3. Weather data

All weather variables were derived from the ERA5-Land reanalysis dataset (Muñoz-Sabater et al., 2021) via Google Earth Engine (Gorelick et al., 2017) to extract daily air temperature (°C) and daily total precipitation (mm). ERA5-Land estimates land-surface meteorological variables by driving a physical land-surface model with ERA5 atmospheric forcing, which is constrained by historical observations (Muñoz-Sabater et al., 2021). To capture temporal variation, we analyzed weather data at two temporal resolutions, including daily resolution for the bat evening emergence time response and a monthly resolution for the guano-yield response. At both daily and monthly resolutions, weather variables were averaged spatially within three ring buffers around the roost (0–10, 10–20, and 20–30 km; Fig. 1a) to determine the extent to which weather at diverse radii of environmental influence predicted bat responses.

### 2.4. Biological response variable

Two biological datasets were used, including bat evening emergence time as a metric of short-term behavioral response, and guano yield as a long-term foraging and demographic metric. Bat evening emergence time was defined as the time at which the first bats began to stream from the cave each evening, the onset of colony emergence (Fig. 1b). The first group of bats to leave the cave initiates the outflow of the rest of the colony and provides a standardized time point that can be recorded each evening consistently (Reichard et al., 2009). The forestry technical officer at KCP NHA recorded the daily evening emergence times of the first bat onset over nine years (2013–2021) as time-out values (hours and minutes) converted to decimal hours (e.g., 17:30–17.50). Since we focused on the onset of bat evening emergence, this metric represents the timing of the earliest-emerging cohort and is not affected by the long tail of the emergence data or by variations in total colony outflow from night to night.

Guano yield, used as a proxy for bat abundance and foraging magnitude, was measured as the total monthly mass (kg) of bat guano commercially harvested from the cave. Guano at KCP NHA has long been collected by the local harvester community under a formal collection concession administered with the resident monastery. Data acquired from local harvesters confirmed that the collection protocol was strictly standardized across the study period. A consistent number of harvesters collected guano from the cave using buckets every Saturday between 4:00 AM and 9:00 AM, and counted and weighed each bucket. Although guano is harvested weekly, these weights were aggregated into monthly yields (1998–2019) to reduce short-term variation. Month-to-month variation in guano yield was interpreted as differences in guano production rather than variations in collection effort. Because guano harvesters remove all accessible guano essentially during each weekly collection rather than sub-sampling, the aggregated monthly value represents all guano deposited during that month rather than multi-year accumulation, providing a colony-integrated index of deposition over the preceding interval (Olival et al., 2015).

Bat reproductive timing is closely linked to resource availability, as female bats require substantial energy during lactation to maximize offspring survival (Speakman, 2008). In tropical ecosystems, insect abundance is positively correlated with rainfall (Nurul-Ain et al., 2017) and typically peaks during the rainy season. In the study area (KCP NHA), the rainy season extends from May to October (Hillman, 2006; Leelapaibul et al., 2005; Utthammachai et al., 2008), resulting in increased insect availability during this period. In addition, insect abundance often peaks approximately one month after periods of maximum rainfall (Cumming and Bernard, 1997). Therefore, bats are expected to initiate pregnancy prior to peak food availability, ensuring that lactation coincides with periods of increasing resource abundance (Furey et al., 2018). To account for this reproductive response to weather, data on the reproductive periods (i.e., April, May, September, and October) were subset for specific seasonal analyses (Furey et al., 2018; Utthammachai et al., 2008).

### 2.5. Seasonality of bat evening emergence time

To measure the colony’s daily evening emergence time, we fitted a two-way analysis of variance (ANOVA) testing the main effects of, and interaction between, observation month and observation year (Emergence ~ Month × Year). A significant interaction term would indicate that the overarching seasonal pattern shifts dynamically from year to year. Model residuals were inspected for approximate normality and homoscedasticity before inference. Post hoc estimated marginal means (EMM) were extracted using the “emmeans” package (Lenth and Piaskowski, 2026) to identify the statistical peaks and lows of the seasonal cycle. All analyses were conducted in the R program (R Core Team, 2026).

### 2.6. Effect of weather on biological responses

To identify the effect of weather on bat evening emergence time and guano yield, we performed an absolute sliding-window analysis using the “climwin” package in R (Bailey and van de Pol, 2016). Absolute windows anchor the candidate windows (i.e., temporal range) to a fixed calendar reference date (i.e., month) for all records. Absolute windows allow specific calendar months for iterative relationship tests between historical weather data and the bat evening emergence time and guano yield response variables across diverse temporal lags and window durations (Bailey and van de Pol, 2016). We selected the best-supported time window using Akaike’s Information Criterion corrected for small sample sizes (AICc) (Haest et al., 2021; van de Pol et al., 2016). For bat evening emergence time, we tested weather lags of up to 90 days, a time window chosen to span the ~1–3-month insect maturation and reproductive cycles likely to influence bat emergence behavior. We compared a baseline null model with models incorporating mean temperature and cumulative precipitation across the three radii of environmental influence. We averaged temperature and cumulative precipitation within each candidate window. We then centered and scaled all weather predictors to unit standard deviation before model fit (Schielzeth, 2010). For guano yield, we set a maximum search window of three months (90 days; matching the bat evening emergence analysis) preceding each monthly guano collection. We analyzed bat evening emergence time in decimal hours and log-transformed monthly guano yield to reduce skewness and stabilize variance using a Gaussian generalized linear model (Quinn and Keough, 2002). Models were ranked by ΔAICc, and the slopes and p-values of the best-supported model (lowest ΔAICc) were extracted to determine the direction and significance of each weather variable.

### 2.7. Long-term guano yield analysis

Proxies of bat abundance were generated using an innovative analysis of monthly guano yield records (1998–2019). From these records, we estimated the bat population (Y) through dimensional analysis following Eq. 1 (Olival et al., 2015).


(1) $$\frac{X\;\text{buckets}}{1\;\text{week}}\times \frac{5.95\;kg\;\text{of guano}}{1\;\text{bucket}}\times \frac{1\;\text{bat per hour}}{0.01075\;g\;\text{of guano}}\times \frac{1\;\text{week}}{168\;\text{hours}}\times \frac{1000\;g}{1\;kg}=Y\;\text{bats}$$

Eq. (1) converts the raw harvest volume (X buckets) into a bat-abundance index using established hourly guano-production rates for this colony (Olival et al., 2015). The resulting monthly abundance is expressed in thousands of individuals. This is equivalent to multiplying by 0.5537 the kilograms of guano generated by bats monthly to infer population sizes. This guano-based demographic approach provides an internally consistent, long-term index of relative bat abundance. We tested for a long-term trend in monthly abundance using a seasonal Mann-Kendall test (Hirsch et al., 1982), which evaluates a monotonic trend while accounting for intra-annual (monthly) seasonality (series beginning October 1998). The standard normal statistic (Z) summarizes the direction and significance of the trend, with negative values indicating a decline and positive values indicating a population increase.

## 3. Results

### 3.1. Seasonal variation

A seasonal pattern of daily bat emergence was observed across the nine-year study period (Fig. 2). The two-way ANOVA confirmed significant effects of month (F_11,3179_=237.34, p < 0.001) and year (F_8,3179_=31.86, p < 0.001), and a significant month × year interaction (F_88,3179_=11.75, p < 0.001), indicating that the seasonal emergence pattern varied among years (Fig. 2a). Post hoc estimated marginal means (EMM) identified June as the month of latest emergence (EMM=18.34 decimal hours; ~18:20 h) and October as the earliest (EMM=17.55 decimal hours; ~17:33 h) (Fig. 2b).

### 3.2. Bat evening emergence

Sliding-window analyses showed that variation in bat evening emergence time tracked sub-seasonal weather lags (Table 1; Fig. 3), with lags of a few days to about three months. For instance, bat evening emergence in April was associated with temperature and precipitation two to six weeks preceding (Table 1). Increased temperatures 16–14 days preceding were associated with later bat evening emergence at 0–10 and 10–20 km (β=0.207 and 0.212, p < 0.001), as was increased temperature over a slightly longer window at 20–30 km (16–12 days preceding; β=0.290, p < 0.001). Increased cumulative precipitation 43–34 days preceding was associated with earlier bat evening emergence, consistently across ring buffers (β=−0.061 at 0–10 km; β=−0.060 at 10–20 km; β=−0.058 at 20–30 km, p < 0.001). In May, increased temperatures 46–44 days preceding were associated with later bat evening emergence at 0–10 and 10–20 km (β=0.207 and 0.212, p < 0.001), and 46–42 days preceding at 20–30 km (β=0.290, p < 0.001). Increased cumulative precipitation was associated with earlier bat evening emergence, consistently across ring buffers (β=−0.048 at 0–10 km, 31–29 days preceding; β=−0.124 at 10–20 km and β=−0.129 at 20–30 km, 29–28 days preceding, p < 0.001) (Table 1). In September, temperature and precipitation acted over lags of roughly one to three months. Increased temperatures 79–76 days preceding were associated with earlier bat evening emergence (β=−0.180 at 0–10 km; β=−0.181 at 10–20 km; β=−0.180 at 20–30 km, p < 0.001), whereas increased cumulative precipitation over a long window 80–33 days preceding was associated with later emergence (β=0.013, p < 0.001), with effect sizes consistent across ring buffers (Table 1). In October, increased temperatures in the preceding 72–70 days were associated with earlier bat evening emergence at 0–10 and 10–20 km (β=−0.169, p < 0.001), while at 20–30 km increased temperatures in the preceding 4–3 days were associated with earlier bat evening emergence (β=−0.136, p < 0.001). Increased cumulative precipitation 57–56 days preceding was associated with earlier bat evening emergence at all ring buffers (β=−0.078 at 0–10 km; β=−0.076 at 10–20 km; β=−0.072 at 20–30 km, p < 0.001) (Table 1).

### 3.3. Guano yield

Monthly guano yield tracked weather conditions at lags of up to three months (Table 2; Fig. 3). Increased temperatures were associated with lower guano yield in April, May, September, and October. Cumulative preceding precipitation was positively associated with guano yield in April and May, negatively associated in September, and not significantly associated in October (Table 2). In April, guano yield decreased with increased temperature in the current month, consistently across ring buffers (β=−0.205 at 0–10 km; β=−0.205 at 10–20 km; β=−0.209 at 20–30 km, p < 0.001). Increased cumulative precipitation over the current and two preceding months was associated with increased guano yield, consistently across ring buffers (β=0.319 at 0–10 km, β=0.319 at 10–20 km, and β=0.321 at 20–30 km, p < 0.001). In May, increased temperature was again associated with lower guano yield in the current month, consistently across ring buffers (β=−0.283 at 0–10 km; β=−0.285 at 10–20 km; β=−0.289 at 20–30 km, p < 0.001). Increased cumulative precipitation over the current and three preceding months was positively associated with guano yield (β=0.260, p < 0.001), with effect sizes consistent across ring buffers (Table 2). In September, guano yield decreased with increased temperature three months preceding, consistently across ring buffers (β=−0.365 at 0–10 km; β=−0.368 at 10–20 km; β=−0.376 at 20–30 km, p < 0.001) and with increased cumulative precipitation two to three months preceding, consistently across ring buffers (β=−0.170 at 0–10 km; β=−0.180 at 10–20 km; β=−0.205 at 20–30 km, p < 0.05) (Table 2). In October, guano yield decreased with increased temperature over the current and preceding months, consistently across ring buffers (β=−0.605 at 0–10 km; β=−0.609 at 10–20 km; β=−0.614 at 20–30 km, p < 0.001), whereas cumulative precipitation showed no significant association (Table 2).

Using guano yield as a proxy to estimate relative bat abundance, we found that the average relative bat abundance was approximately 6.82 million over the study period. The estimated relative bat abundance ranged from a maximum of 16.918 million in July 2000 to a minimum of 1.861 million in May 2016 (Fig. 4). A linear regression of the relative bat abundance suggested a 56.62% decline (β=−0.691, R^2^=0.34, p < 0.001). A seasonal Mann-Kendall trend analysis confirmed a significant, long-term monotonic decrease in relative bat abundance over the same two decades (Z = −11.585, p < 0.001; Fig. 4).

## 4. Discussion

Our study of a wrinkle-lipped free-tailed bat colony at KCP NHA presents three main findings. First, the evening emergence in wrinkle-lipped free-tailed bats suggests behavioral response in the form of modified behavior linked to sub-seasonal weather variation at lags of a few days to about three months, consistent with behavioral plasticity. Second, monthly guano yield was linked to weather variation within the preceding three months, depending on season. Third, relative bat abundance shows a sustained decline across two decades. Together, these findings illustrate how weather variation may modulate behavior and demographic parameters of an insectivorous bat.

### 4.1. Effects of weather on bat evening emergence

Bat evening emergence reflects a nightly trade-off between the energetic benefits of early foraging and the lethal risks of diurnal predation (Duvergé et al., 2000). Our nine-year bat evening emergence dataset suggests that although the wrinkle-lipped free-tailed bat follows a consistent circannual rhythm of foraging, emergence timing can change in response to weather variation (i.e., behavioral plasticity). Mean monthly bat evening emergence was latest in June and earliest in October. The bat evening emergence time shift (~47 min) may reflect the seasonal changes in local sunset time (Reichard et al., 2009). Alternatively, variation in bat evening emergence time could also be independent of local weather and instead reflect the bimodal polyoestry of the wrinkle-lipped free-tailed bat, with parturition occurring primarily in April and October (Furey et al., 2018). Pregnant females experience increased wing loading and reduced flight maneuverability, which may force them to emerge later, potentially to avoid visually orienting raptors (Reichard et al., 2009). Conversely, lactating females face extreme metabolic stress for milk production and may emerge earlier to maximize caloric intake during the dusk peak of aerial insect activity (Duvergé et al., 2000; Reichard et al., 2009). This variation in bat evening emergence time could be a case of behavioral plasticity of open-space foragers that rely on fast flight to increase foraging success or reduce predation risk or both (Thomas and Jacobs, 2013).

The wrinkle-lipped free-tailed bat in the Oriental Region remains far less studied than its relative, the Brazilian free-tailed bat (*Tadarida brasiliensis*), in the Nearctic and Neotropical Regions. Nevertheless, the two species are expected to follow broadly parallel patterns given their similar ecology (Cleveland et al., 2006; Horn and Kunz, 2008; McCracken et al., 2008; Srilopan et al., 2018). Both bat species form enormous cave colonies of up to millions of individuals, forage in open airspace at high altitude, and capitalize on seasonal pulses of migratory agricultural pest insects, delivering substantial pest-control services (McCracken et al., 2008; Wanger et al., 2014). These two bat species, however, differ in ecological setting and diet. North American populations of the Brazilian free-tailed bat are migratory and feed heavily on moths, whereas the wrinkle-lipped free-tailed bat is a resident of the tropical monsoon zone of Southeast Asia and feeds predominantly on hemipteran planthoppers of irrigated rice and provides a local pest-control service (Cleveland et al., 2006; Leelapaibul et al., 2005; McCracken et al., 2008; Srilopan et al., 2018). In the study area, rice is grown year-round and harvested asynchronously, and planthoppers become a major component of the wrinkle-lipped free-tailed bat’s diet during their mass emigration at the February-March and November-December rice harvests (Leelapaibul et al., 2005). Weather effects on the study colony and the bat evening emergence time may therefore act partly through the planthopper phenology.

The sliding-window analysis revealed time-lagged weather cues associated with day-to-day variation in bat evening emergence time, with the most influential cues preceding emergence by lags of a few days to about three months.

Temperature produced contrasting seasonal effects on the evening emergence times of wrinkle-lipped free-tailed bats. For example, warmth in mid-March, about two weeks before April and six to seven weeks before May, was associated with later bat evening emergence at all ring buffers. By September and October, the temperature relationship reversed, with increased temperatures a few days to three months preceding associated with earlier bat evening emergence. Because the wrinkle-lipped free-tailed bat has two primary parturition peaks (April and October) (Furey et al., 2018), reproductive state alone could not explain why warmth predicts later emergence in April but earlier bat evening emergence in October. An alternative explanation would be a seasonal shift in the temperature-insect relationship, whereby warm conditions early in the year delay the development of the insect prey base, whereas warm conditions late in the wet season bring prey pulses forward as described elsewhere (Piyaphongkul et al., 2012a, 2012b; Skendžić et al., 2021). A second potential explanation is that late-gestation constraints, such as increased wing loading around the April parturition peak, push bat evening emergence later independently of prey availability (Furey et al., 2018; a response documented in other large molossid colonies, such as the Brazilian free-tailed bat; Reichard et al., 2009). Distinguishing these mechanisms will require complementary data on insect phenology and on the reproductive condition of emerging females, which were not available to us.

In contrast to temperature, increased cumulative precipitation was associated with earlier bat evening emergence in April, May, and October, but with later emergence in September. In September, increased cumulative precipitation over a broad window of one to nearly three months preceding was associated with slightly later bat evening emergence, a pattern that coincided with the response of guano yield (see below *Effects of weather on guano yield*). During April, at the onset of the hot season, increased cumulative precipitation about five to six weeks preceding was associated with earlier bat evening emergence. Rather than an immediate response to same-night conditions, this temporal lag in bat evening emergence is consistent with weather acting on the development of the insect prey base, as insect abundance in seasonal tropical systems peaks about a month after peak rainfall (Cumming and Bernard, 1997), and the first rains after the dry season can rapidly increase insect herbivore abundance (Novais et al., 2019). Similar lagged responses occurred in May and October, when increased cumulative precipitation approximately four and eight weeks preceding was associated with significantly earlier bat evening emergence. These lags could reflect the time needed for insects to develop after rainfall and increase in abundance.

### 4.2. Effects of weather on guano yield

Monthly guano yield combines foraging success and colony size across the whole month (Furey and Racey, 2016). Because GPS-tracked wrinkle-lipped free-tailed bats foraged a median maximum distance of 26 km from the roost (Srilopan et al., 2025), our 10–30 km ring buffers encompass much of their foraging range, and the near-identical coefficients across the buffer sizes suggest the associations were consistent across spatial scales.

Increased temperatures were associated with reduced guano yield in April, May, September, and October. The reduction in guano yield linked to increased temperature may be owing to heat-driven reductions in insect availability (Piyaphongkul et al., 2012b). Alternatively, higher thermoregulatory costs for the colony during warmer periods may induce variation in guano yields (Walker et al., 2026). This consistent sensitivity to heat suggests that warming could reduce foraging returns across the season.

In contrast to the consistent effects of temperature, responses to precipitation depended on season. Guano yield in April and May was most strongly associated with increased cumulative precipitation over the preceding two to three months, a temporal lag consistent with the delay between rainfall, insect development, and peak prey availability in seasonal tropical systems (Cumming and Bernard, 1997). In the study area, this rainfall signal is consistent with the agricultural phenology where the brown planthopper (*Nilaparvata lugens*) and the white-backed planthopper (*Sogatella furcifera*) flourish (Srilopan et al., 2018). The wrinkle-lipped free-tailed bat intercepts migratory swarms of prey insects hundreds of meters above the agricultural canopy (Srilopan et al., 2025), which could provide a plausible route from rainfall to guano production. In September, however, increased cumulative precipitation two to three months preceding was associated with lower guano yield, and increased cumulative precipitation over a similar window was associated with later bat evening emergence. Both windows fall largely within June to August, a rainy-season period of high rainfall in our study area (Leelapaibul et al., 2005). Whereas rainfall at the onset of the hot season in April and May may promote prey pulses (Novais et al., 2019), additional rainfall once the wet season is well established may limit rather than support foraging in September. Heavy rainfall could hinder the flight and dispersal of planthoppers (Chen et al., 2019), potentially reducing the airborne prey available to high-flying bats, whereas wet fur doubles the energetic cost of bat flight and may discourage foraging during prolonged rain (Voigt et al., 2011).

Bat evening emergence also responded to precipitation at lags of about four to eight weeks, suggesting that lagged precipitation effects are shared by behavioral and colony-level responses, consistent with lagged climate sensitivity reported across taxa (Thackeray et al., 2016; van de Pol et al., 2016). Together, these results suggest that rainfall benefits for the wrinkle-lipped free-tailed bat colony occur mainly in the months when it supports agricultural insect outbreaks, and that warming could erode these foraging advantages even when seasonal rainfall remains favorable.

### 4.3. Relative bat abundance

Our data revealed a sustained decline in relative bat abundance estimates derived from guano yield across two decades. This decline in relative bat abundance suggests that short-term behavioral plasticity, such as shifts in emergence time, may not be sufficient to maintain guano production (Radchuk et al., 2019). Climate change can alter the rainfall regimes that govern plant productivity, resulting in increases or decreases in insect abundance (Skendžić et al., 2021). By disrupting the synchrony among rainfall, rice growth, planthopper emergence, and bat reproduction, future climate temperature and altered rainfall could progressively decouple prey supply from bat foraging (Festa et al., 2023). Because wrinkle-lipped free-tailed bats, as insectivores, help control insect pests in rice agroecosystems (Tuneu-Corral et al., 2024; Wanger et al., 2014), a continued decline in this colony may reduce the pest-control services it provides.

## 5. Conclusion

The large wrinkle-lipped free-tailed bat colony at KCP NHA in Thailand reveals that bat evening emergence time varied along gradients of increasing temperature and precipitation with lags of a few days to about three months. Monthly guano yield decreased with increased temperature at lags of up to three months, while responses to increased precipitation varied seasonally, from positive in April-May to negative in September. Estimates of relative bat abundance derived from guano yield across two decades revealed a steep decline (56.62%). Short-term foraging behavior adjustments were linked to local weather variation, but long-term colony-level responses, such as guano yield and relative abundance, had stronger responses to temperature and precipitation changes. The persistence of bat colonies may therefore be impacted by regional climatic variation influencing insect prey availability, bat behavior, and bat demography.

## Figures and Tables

**Fig. 1.**
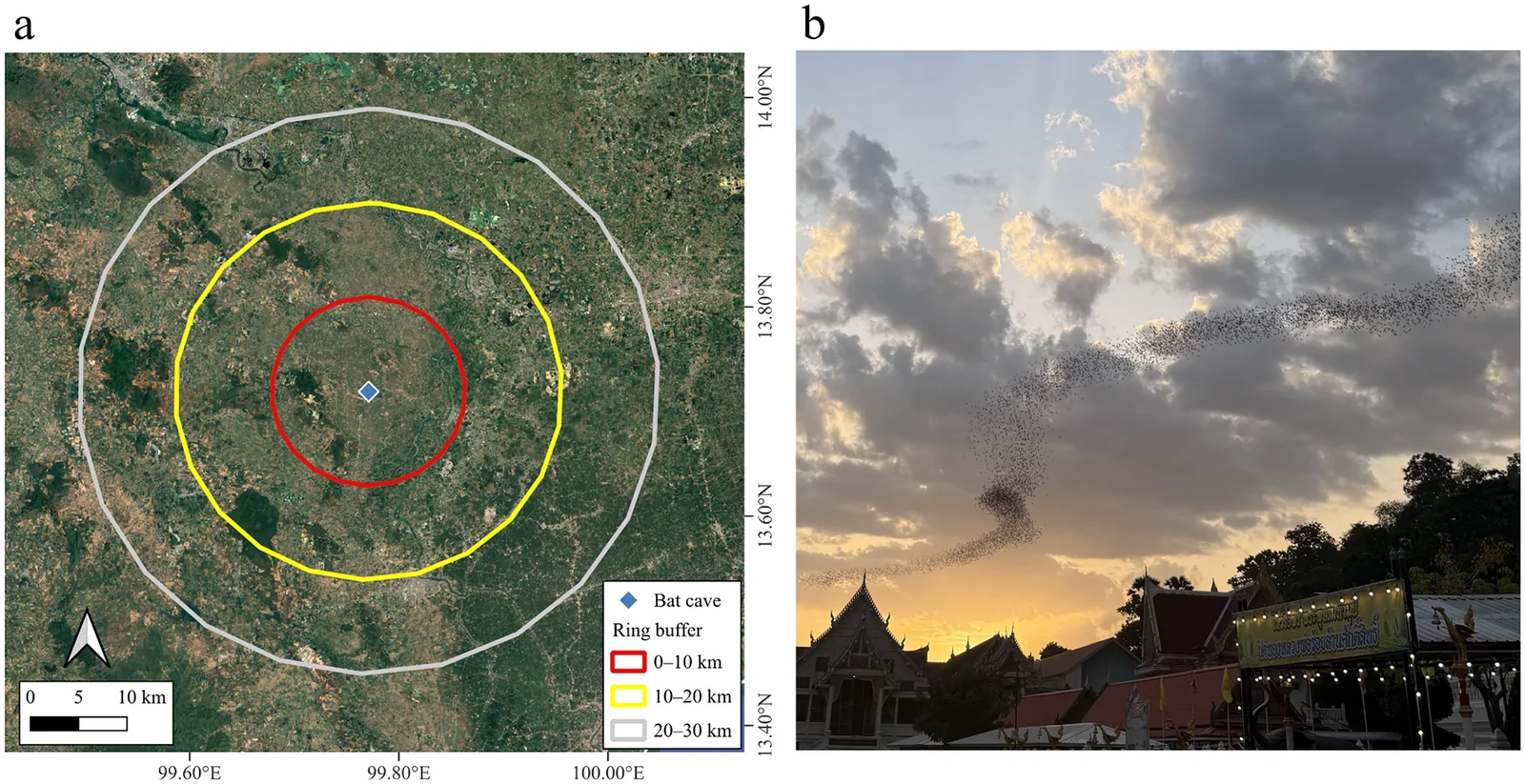
Map of the Khao Chong Phran Non-hunting Area (KCP NHA) study site in Ratchaburi province, Thailand. (a) Concentric ring buffers (0–10 km, 10–20 km, and 20–30 km) surrounding the central bat cave (blue diamond) denote the multiscale spatial zones from which weather data (temperature and precipitation) were extracted. The study cave is located at 13.719° N, 99.771° E. Satellite background imagery was generated in QGIS. (b) Evening emergence of the wrinkle-lipped free-tailed bat (*Mops plicatus*) colony at KCP NHA. Photograph taken by Paanwaris Paansri on July 18, 2026, at 6:33 PM.

**Fig. 2.**
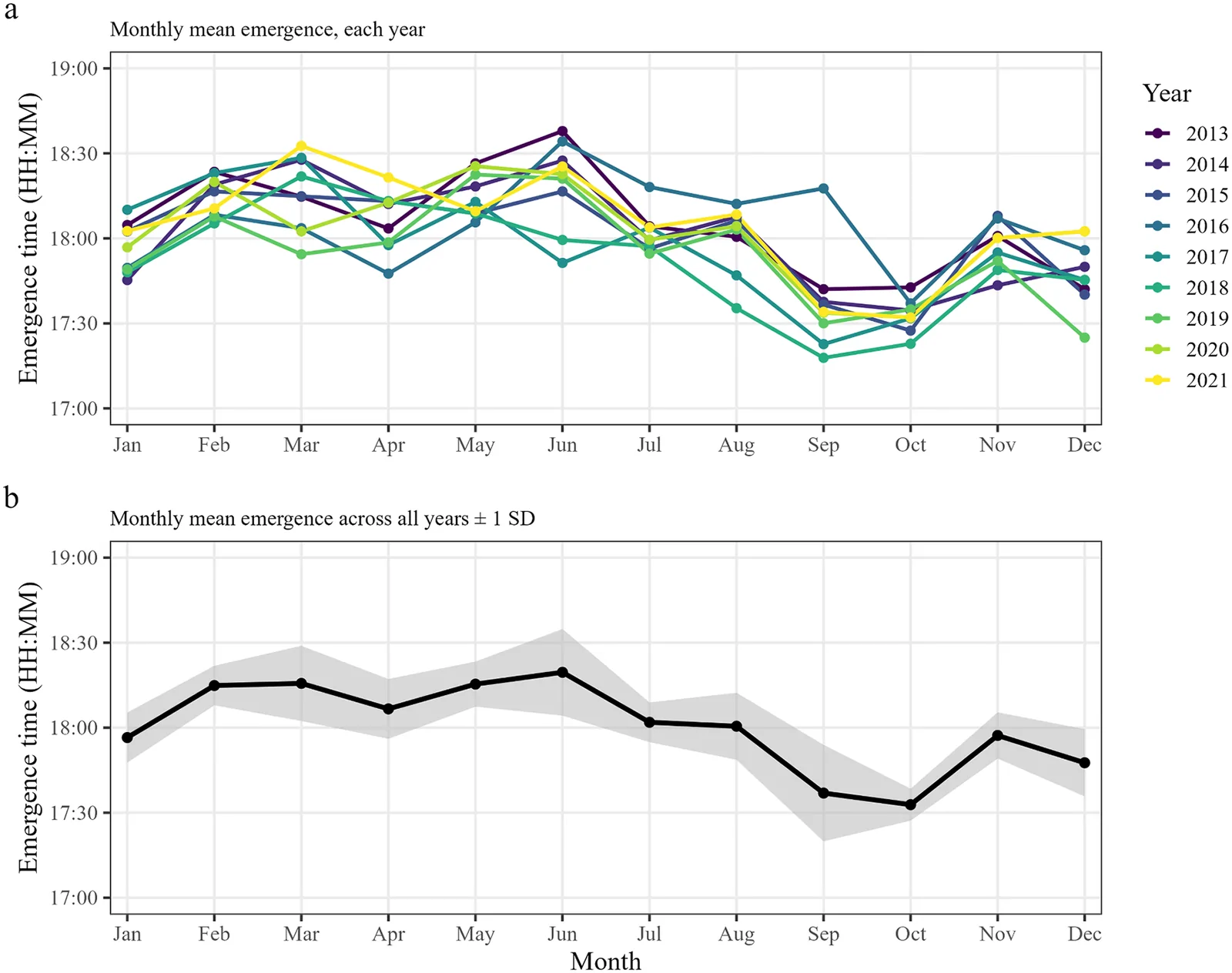
Seasonal pattern of evening emergence time in the wrinkle-lipped free-tailed bat colony (2013–2021). (a) Monthly mean emergence time for each year, with color denoting year (2013, dark purple, through 2021, yellow). (b) Mean monthly emergence time across all years (black line) with a shaded ±1 SD band showing among-year variation. The x-axis is the month of the year (January–December) and the y-axis is emergence time (HH:MM).

**Fig. 3.**
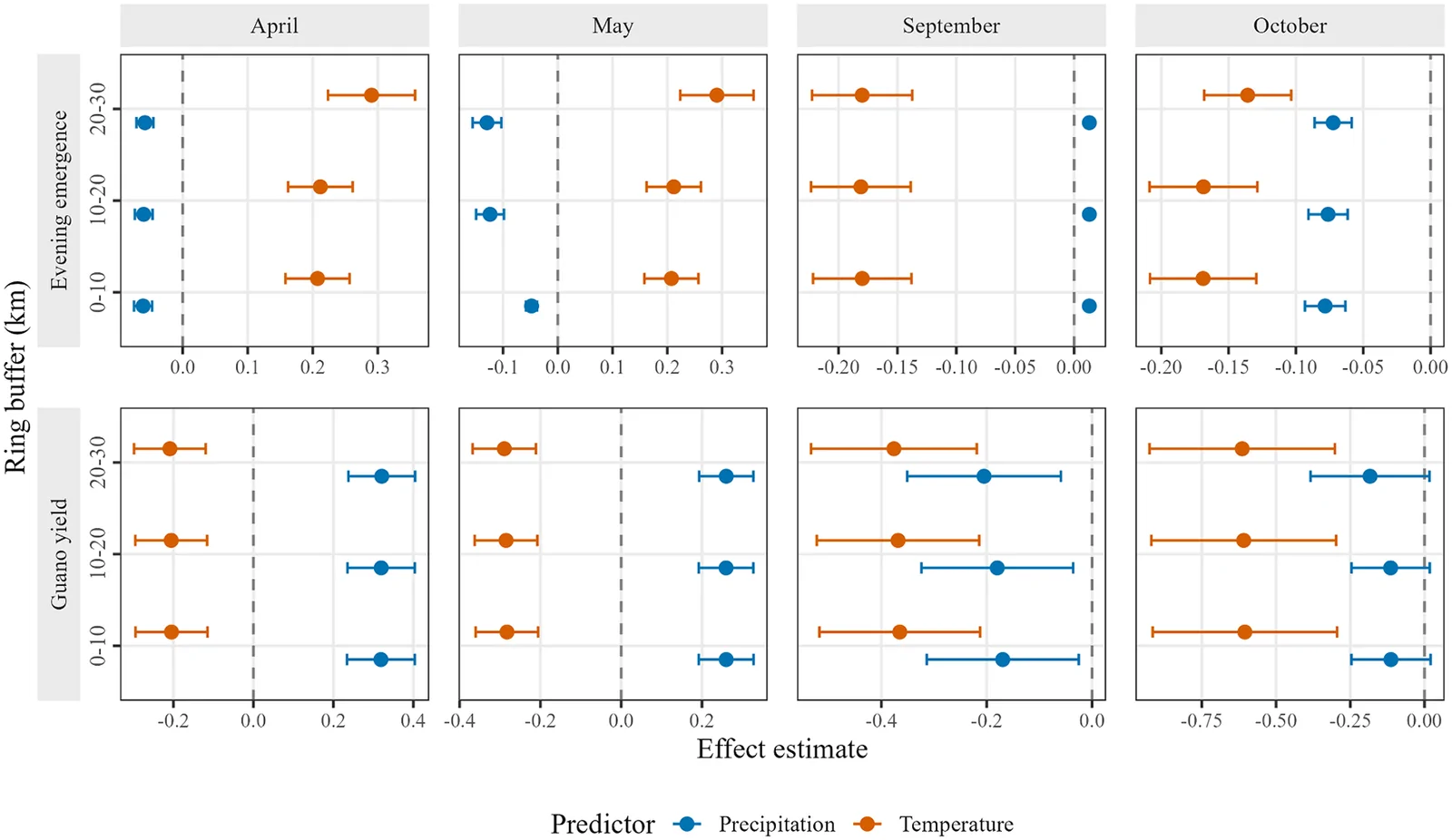
Effects of preceding weather on bat responses, from the best-supported sliding-window models. Point: Model coefficient (effect estimate) for temperature (orange) or precipitation (blue) aggregated within the 0–10, 10–20, and 20–30 km ring buffers. Horizontal bars: 95% confidence intervals. Figures show that all estimates were significant (p < 0.05) except October precipitation in the guano-yield models. Columns: Four focal months (April, May, September, October) were selected due to data on the reproductive periods. Top row: Daily emergence response. Bottom row: Monthly guano-yield response. Dashed vertical line: Null effect (0), where left points indicate negative association (earlier bat evening emergence or lower guano yield) and right points indicate positive association (later bat evening emergence or higher guano yield) in response to increased temperature or increased cumulative precipitation.

**Fig. 4.**
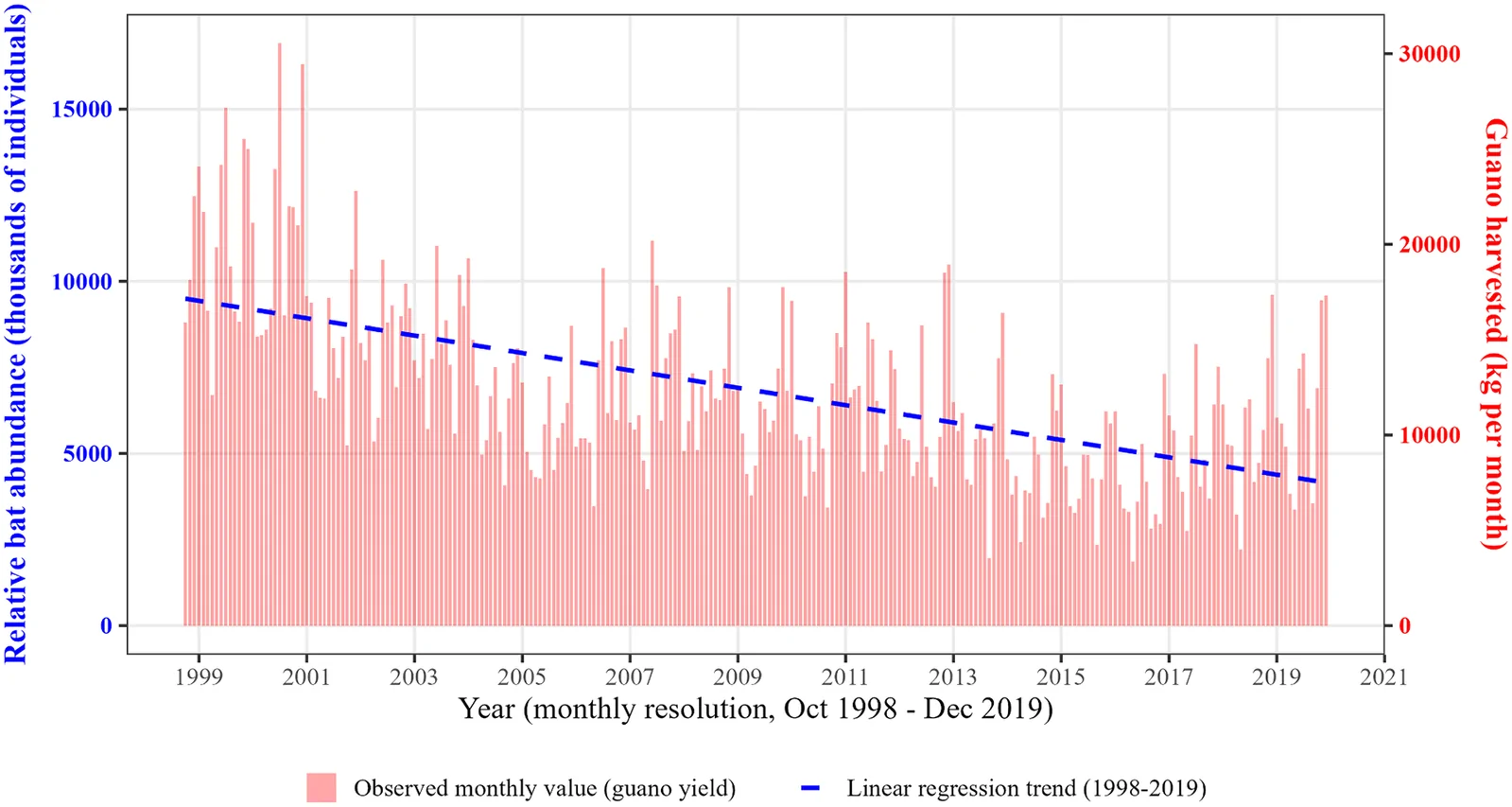
Long-term estimate of the relative abundance of the studied wrinkle-lipped free-tailed bat colony from 1998 to 2019. X-axis: time, in calendar years at a monthly resolution (October 1998-December 2019; n = 255). Left y-axis (y1, blue): Estimated relative bat abundance in thousands of individuals. Dashed blue line: Ordinary least-squares linear trend shows a 56.62% decline (β=−0.691, R^2^=0.34, p < 0.001). Right y-axis (y2, red): Equivalent monthly guano yield in kilograms. Red bars: Observed monthly values. Results of a seasonal Mann-Kendall test (Z = −11.585, p < 0.001) indicate a statistically significant long-term downward trend over the 22-year monitoring period.

**Table 1:** The effect of weather windows for predictors of the daily evening emergence time of the wrinkle-lipped free-tailed bat. For each calendar month and weather predictor (evaluated at 0–10, 10–20, and 20–30 km ring buffers), the table reports the best-supported weather window (days preceding) identified via sliding-window analysis based on ΔAICc. Positive slope estimates (β) represent later emergence, whereas negative values represent earlier bat evening emergence.

Month	Weather Predictor	ΔAICc	Window (Days preceding)	Slope Estimate (β)	Std. Error	p-value
April	Temperature (0–10 km)	−65.27	16–14	0.207	0.025	< 0.001
	Temperature (10–20 km)	−67.22	16–14	0.212	0.025	< 0.001
	Temperature (20–30 km)	−69.52	16–12	0.290	0.034	< 0.001
	Precipitation (0–10 km)	−70.61	43–34	−0.061	0.007	< 0.001
	Precipitation (10–20 km)	−71.92	43–34	−0.060	0.007	< 0.001
	Precipitation (20–30 km)	−73.76	43–34	−0.058	0.007	< 0.001
May	Temperature (0–10 km)	−65.27	46–44	0.207	0.025	< 0.001
	Temperature (10–20 km)	−67.22	46–44	0.212	0.025	< 0.001
	Temperature (20–30 km)	−69.52	46–42	0.290	0.034	< 0.001
	Precipitation (0–10 km)	−85.61	31–29	−0.048	0.005	< 0.001
	Precipitation (10–20 km)	−86.98	29–28	−0.124	0.013	< 0.001
	Precipitation (20–30 km)	−89.73	29–28	−0.129	0.013	< 0.001
September	Temperature (0–10 km)	−68.47	79–76	−0.180	0.021	< 0.001
	Temperature (10–20 km)	−67.57	79–76	−0.181	0.022	< 0.001
	Temperature (20–30 km)	−66.14	79–76	−0.180	0.022	< 0.001
	Precipitation (0–10 km)	−113.54	80–33	0.013	0.001	< 0.001
	Precipitation (10–20 km)	−114.02	80–33	0.013	0.001	< 0.001
	Precipitation (20–30 km)	−114.54	80–33	0.013	0.001	< 0.001
October	Temperature (0–10 km)	−67.51	72–70	−0.169	0.020	< 0.001
	Temperature (10–20 km)	−65.45	72–70	−0.169	0.020	< 0.001
	Temperature (20–30 km)	−65.27	4–3	−0.136	0.016	< 0.001
	Precipitation (0–10 km)	−100.69	57–56	−0.078	0.008	< 0.001
	Precipitation (10–20 km)	−101.26	57–56	−0.076	0.007	< 0.001
	Precipitation (20–30 km)	−102.31	57–56	−0.072	0.007	< 0.001

**Table 2:** The effect of weather windows for predictors of monthly guano yield of the wrinkle-lipped free-tailed bat colony. For each calendar month and weather predictor (evaluated at 0–10, 10–20, and 20–30 km ring buffers), the table reports the best-supported weather window identified via sliding-window analysis based on ΔAICc. Positive slope estimates (β) indicate an increase in guano yield, whereas negative slopes indicate a decrease in guano yield.

Month	Weather Predictor	ΔAICc	Window (Months Preceding)	Slope Estimate (β)	Std. Error	p-value
April	Temperature (0–10 km)	−17.25	0–0	−0.205	0.046	< 0.001
	Temperature (10–20 km)	−17.44	0–0	−0.205	0.046	< 0.001
	Temperature (20–30 km)	−18.2	0–0	−0.209	0.046	< 0.001
	Precipitation (0–10 km)	−47.59	2–0	0.319	0.043	< 0.001
	Precipitation (10–20 km)	−48.32	2–0	0.319	0.043	< 0.001
	Precipitation (20–30 km)	−49.62	2–0	0.321	0.043	< 0.001
May	Temperature (0–10 km)	−45.13	0–0	−0.283	0.039	< 0.001
	Temperature (10–20 km)	−45.39	0–0	−0.285	0.040	< 0.001
	Temperature (20–30 km)	−45.81	0–0	−0.289	0.040	< 0.001
	Precipitation (0–10 km)	−49.26	3–0	0.260	0.035	< 0.001
	Precipitation (10–20 km)	−49.61	3–0	0.260	0.034	< 0.001
	Precipitation (20–30 km)	−50.4	3–0	0.260	0.034	< 0.001
September	Temperature (0–10 km)	−19.21	3–3	−0.365	0.078	< 0.001
	Temperature (10–20 km)	−19.15	3–3	−0.368	0.079	< 0.001
	Temperature (20–30 km)	−19.17	3–3	−0.376	0.080	< 0.001
	Precipitation (0–10 km)	−3.25	3–2	−0.170	0.074	0.022
	Precipitation (10–20 km)	−3.93	3–2	−0.180	0.073	0.015
	Precipitation (20–30 km)	−5.5	3–2	−0.205	0.074	0.006
October	Temperature (0–10 km)	−12.24	1–0	−0.605	0.158	< 0.001
	Temperature (10–20 km)	−12.36	1–0	−0.609	0.159	< 0.001
	Temperature (20–30 km)	−12.52	1–0	−0.614	0.159	< 0.001
	Precipitation (0–10 km)	−0.72	1–1	−0.113	0.068	0.098
	Precipitation (10–20 km)	−0.83	1–1	−0.114	0.067	0.091
	Precipitation (20–30 km)	−1.18	3–1	−0.183	0.102	0.074

## Data Availability

https://doi.org/10.5281/zenodo.22666741
